# When function mirrors structure: how slow waves are shaped by cortical layers

**DOI:** 10.1186/1471-2202-16-S1-P161

**Published:** 2015-12-18

**Authors:** Cristiano Capone, Beatriz Rebollo, Alberto Muñoz-Cespédes, Paolo Del Giudice, Maria Victoria Sanchez-Vives, Maurizio Mattia

**Affiliations:** 1 PhD Program in Physics, Sapienza University, Rome, Italy; 2 Istituto Superiore di Sanità, Rome, Italy; 3 IDIBAPS, Barcelona, Spain; 4 Universidad Complutense de Madrid, Madrid, Spain..; 5 ICREA, Barcelona, Spain

## 

Neuronal spontaneous activity can provide valuable information on the functional structure of the underlying neuronal network. We focused on the spontaneous slow-wave activity that is generated and propagated in cortical slices, aiming at relating their spatio-temporal organization with the laminar structure of the tissue. We extracted multi-unit activities from simultaneous recordings obtained by means of an array of 16 electrodes covering the slice. Although each site displayed alternation between high-firing (Up) and almost quiescent (Down) states, slow oscillations (SO) were not spatially homogeneous, showing layer-dependent state durations and maximum firing rates. A distribution of time lags between electrodes in the onset and offset of Up states reflected a propagating activity wave. We found different propagation modes in terms of velocity, direction, wavefront shape (see Figure 1A), spatial extension and site of origin. Despite such variability, at the level of single waves, we consistently found that the head of the wavefront had a spatial distribution forming a strip almost parallel to the cortical surface (see Figure 1B black line), at a depth compatible with layer 4 and 5 (L4 and L5). Moreover, this strip widely overlapped with the region where Up states had the maximum duration and magnitude (see Figure 1B respectively green and blue line), a region possibly corresponding to the most excitable part of the network. To test such hypothesis, we tried to reproduce the properties of this strip using a large scale simulation of a slice model, with oscillating cortical modules of spiking neurons arranged in a 2D lattice with nearest neighbor connectivity. In this homogeneous medium we implemented a non-monotonic gradient in the connectivity level (see Figure 1C), aiming to embody the physiological differences in layer excitability, and setting an overexcited strip (dark circles). We found that the connectivity level increase was not trivially related to an increase of the speed of wave propagation, rather this parameter has to be chosen in an optimal range (see Figure 1C-D) in order to reproduce propagating patterns compatible with the experiments. Optimal networks were those having a metastable Down state in which finite-size fluctuations were capable to prime Down-to-Up transitions. In addition to this, we further predicted that SO features like maximum firing rate and longest Up state durations should be found at the same excitable strip.

**Figure 1:**
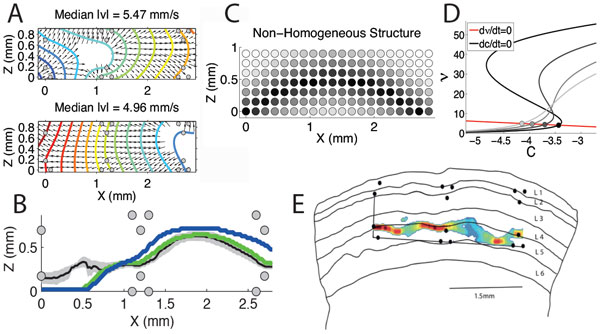
**A. Wavefronts for 2 modes of propagation**. B. Average strips where wavefronts propagate earlier (black), and where Up states have maximum duration (green) and magnitude (blue). C. Modulation of the connectivity parameter in the model. D. Nullclines under mean-field approximation varying levels of connectivity. and C are average firing rate and fatigue level, respectively. Circles, fixed points. Dark to light gray, different excitability levels as in C, respectively. E. Example match between strip of early wave propagation and slice's layers.

## Conclusions

As the model predicted, we found that strips of early wave propagation reliably overlapped with the regions where maximum Up state duration and firing activity occurred, strengthening the duality between spontaneous activity and network structure. Finally, we matched the excitable strips with the slice cortical layers as identified by histology, finding a reliable overlap between such strips and L4 and L5 (see Figure 1E).

